# Knowledge, attitudes and practice survey about antimicrobial resistance and prescribing among physicians in a hospital setting in Lima, Peru

**DOI:** 10.1186/1472-6904-11-18

**Published:** 2011-11-15

**Authors:** Coralith García, Liz P Llamocca, Krystel García, Aimee Jiménez, Frine Samalvides, Eduardo Gotuzzo, Jan Jacobs

**Affiliations:** 1 Instituto de Medicina Tropical Alexander von Humboldt, Universidad Peruana Cayetano Heredia, Lima, Perú; 2 Hospital Nacional Cayetano Heredia, Lima, Perú; 3 Institute of Tropical Medicine Antwerp, Antwerp, Belgium; 4 Department of Medical Microbiology, Faculty of Health, Life Sciences and Medicine, Maastricht University, The Netherlands

**Keywords:** Antimicrobial resistance - Antimicrobial use - Knowledge, attitude and practice survey

## Abstract

**Background:**

Misuse of antimicrobials (AMs) and antimicrobial resistance (AMR) are global concerns. The present study evaluated knowledge, attitudes and practices about AMR and AM prescribing among medical doctors in two large public hospitals in Lima, Peru, a middle-income country.

**Methods:**

Cross-sectional study using a self-administered questionnaire

**Results:**

A total of 256 participants completed the questionnaire (response rate 82%). Theoretical knowledge was good (mean score of 6 ± 1.3 on 7 questions) in contrast to poor awareness (< 33%) of local AMR rates of key-pathogens. Participants strongly agreed that AMR is a problem worldwide (70%) and in Peru (65%), but less in their own practice (22%). AM overuse was perceived both for the community (96%) and the hospital settings (90%). Patients' pressure to prescribing AMs was considered as contributing to AM overuse in the community (72%) more than in the hospital setting (50%). Confidence among AM prescribing was higher among attending physicians (82%) compared to residents (30%, p < 0.001%). Sources of information considered as very useful/useful included pocket-based AM prescribing guidelines (69%) and internet sources (62%). Fifty seven percent of participants regarded AMs in their hospitals to be of poor quality. Participants requested more AM prescribing educational programs (96%) and local AM guidelines (92%).

**Conclusions:**

This survey revealed topics to address during future AM prescribing interventions such as dissemination of information about local AMR rates, promoting confidence in the quality of locally available AMs, redaction and dissemination of local AM guidelines and addressing the general public, and exploring the possibilities of internet-based training.

## Background

Antimicrobial resistance (AMR) is a worldwide problem preferentially affecting low- and middle income countries [1,2]. Two main contributing factors are (i) excessive use of antimicrobials (AMs) adding to an increased selection pressure and (ii) insufficient infection control policies favouring the spread of resistant microorganisms [3]. Patients who receive AMs have an increased risk of acquiring infection from resistant microorganisms [4] and such infections may be associated with increased mortality and morbidity [5,6]. Reduction in AM use is a cornerstone in the containment of AMR and can be addressed through changes in prescribing behaviour. Therefore, knowledge about the driving forces behind AM prescription is needed, and such information can be obtained by means of so-called KAP-surveys (knowledge, attitudes and practice surveys). KAP-surveys about antimicrobial resistance have been conducted among medical doctors in the community setting, but at the time of submission, only five have been reported from the hospital setting, including only one from a middle-income country [7-11] (Table 1).

**Table 1:** Knowledge, attitudes and practices (KAP)-surveys about AMR in the hospital setting as reported in the English literature

Author	Country	Participants	Main findings
Pulcini *et al*., 2010	Scotland, France	Junior doctors (n = 139)	95% agreed AMR is a national problem, 63% agreed so for their own clinical practice. Only 26% knew the correct local prevalence of methicillin-resistant *S. aureus*
Guerra *et al*., 2007	Brazil	Mainly residents (n = 310)	95% agreed AMR is a problem and 87% that AMs are overprescribed
Giblin *et al*., 2004	USA	Health care workers (n = 117*)	95% agreed that AMR is a national problem, 65% agreed for in their own practice
Srinisavan *et al*., 2004	USA	House-staff physicians (other than paediatricians) (n = 179)	88% agreed that AMs are overused in general, 72% agreed so for their own hospital
Wester *et al*. 2002	USA	Internal medicine doctors (n = 490)	87% considered AMR as very important national problem

AMR: antimicrobial resistance AM: antimicrobial

* 33% of participants were physicians

The present study shows the results of a KAP-survey about AMR and AM prescribing among medical doctors from two hospitals in Lima, Peru. The survey was conducted in order to explore and target educational interventions about AM prescribing.

## Methods

### Study design, period and setting

The study consisted of a cross sectional survey of physicians from two public hospitals, Cayetano Heredia (CHH) and Arzobispo Loayza (ALH) during January 2009. Both hospitals are tertiary-level, teaching hospitals located in urban areas of Lima with 423 and 788 patient beds respectively.

### Participants and survey instrument

A self-administered questionnaire was distributed in both hospitals among residents (*i.e*. physicians in training) and attending physicians (*i.e*. staff physicians after completion of training and specialization). Medical doctors from psychiatry, radiology, ophthalmology and anaesthesiology were not included as they do not routinely prescribe AMs. Questionnaires were distributed on site during working hours and participants were asked to respond immediately. There was no incentive for subjects to participate and no reminders were supplied. The questionnaire content was based on a previous survey described in the U.S. and adapted to the Peruvian system [9]. Prior to release, it was reviewed by a team of six Peruvian infectious diseases physicians to assess the relevance and wording of the questions as well as accuracy of the translation into Spanish. The 38-item questionnaire addressed the professional profile of the participants and frequency of AM prescription (5 questions), their awareness about the current scope of AMR (6 questions), sources of information and continuing education about AMs (2 questions), confidence and seeking inputs (5 questions), factors influencing decisions around AM prescription (5 questions) and the acceptability and appropriateness of potential interventions (6 questions) (Additional file 1). Questions used a 4 or 5-point Likert scale (which included answers ranging from "strongly agree" to "strongly disagree", from "very useful" to "not useful at all" and from "always" to "never"). The survey also included seven questions that assessed basic knowledge about the clinical indications, spectrum, administration and pharmacology of AMs. Three case-based questions addressed the choice of AMs for treating acute diarrhoea, an upper respiratory tract infection and sepsis in a patient with impaired renal function; one question addressed safety of AMs during pregnancy, and three questions addressed the spectrum of AMs and their ability to cross the blood-brain barrier. Finally, in order to evaluate physician awareness about AMR rates within local hospitals, participants were asked to estimate the proportion of *Klebsiella pneumoniae *resistance to cephalosporins and *Pseudomonas aeruginosa *resistance to ciprofloxacin (answer options "20% or less", "20%-50%", "more than 50%" or "don't know"). The true rate was obtained from a surveillance study on AMR in Lima hospitals in 2008.

### Ethical clearance

The study was approved by the Institutional Review Board from Universidad Peruana Cayetano Heredia, Lima, Peru, and by the Ethical Committees in each hospital. Based on the anonymous nature of the collected data, informed consent form was not taken.

### Statistical analysis

A sample size of 234 was calculated using the Epi Info 3.5.3 software (CDC, Atlanta, USA) considering a total population of 1050 physicians and the expected correct answer on the questions about knowledge of local AMR rates. This was set at 27% according to a previous publication [7] and a 95% of confidence level was applied. Participants were not sampled randomly. Proportions were calculated for categorical variables and their significance assessed by the Chi square or Fisher's exact test. Means and standard deviations were calculated for continuous variables. Unless otherwise stated, we used Likert items by combining the data into two categories, "strongly agree/agree", "very useful/useful" and "very confident/confident" versus the remaining options of the scale. Data were analysed with the software STATA 10.1 (Statacorp, Texas, USA).

## Results

### Demographics and professional profile

A total of 260/317 physicians filled in the questionnaire (response rate 82%). Four were excluded since they did not specify which department they were affiliated to. The vast majority of participants (97%) agreed that knowledge about AMs and their adequate use are important in their daily work and 49% declared to prescribe AMs more than once a day. Table 2 gives an overview of the professional profile of the 256 participants; the profiles were similar within the two hospitals except for the proportion of surgeons. Unless otherwise stated, there were no significant differences between the participants belonging to different professional categories and levels, departments or hospitals for the results presented below.

**Table 2:** Professional profile of the participants in the two hospitals of Lima, Peru

Characteristic	Cayetano Heredia hospital (n = 132)	Arzobispo Loayza Hospital (n = 124)	Total (n = 256)
**Working time in hospitals**			
0 - 4 years	74 (56)	66 (53)	140 (55)
≥ 5 years	58 (44)	58 (47)	116 (45)
Hospital department			
Medicine	66 (50.0)	76 (61)	142 (55)
Surgery	39 (30)*	14 (11)*	53 (21)
O&B	15 (11)	17 (14)	32 (13)
Paediatrics	12 (9)	17 (14)	29 (11)
Position			
Resident (in training)	64 (48)	71 (57)	135 (53)
Attending physician	68 (52)	53 (43)	121 (47)

Data represent numbers (%).

*p < 0.05 O&B: Obstetrics and Gynaecology

### Knowledge on AM use and AMR rates

The average score to the questions regarding knowledge of AMs was 6 out of 7(SD ± 1.3). For the case-based questions about acute diarrhoea and upper respiratory tract infection, the vast majority of participants agreed that there was no need to start an AM (238, 93% and 194, 76%, respectively). The knowledge about the need to reduce the dose of AM in a patient with severe renal impairment was assessed by presenting a sepsis case where ceftriaxone and gentamicin were prescribed. About three quarters (n = 194, 76%) correctly identified that AMs would need to be reduced in this case. Furthermore, nearly all participants (n = 250, 99%) correctly replied that metronidazole has activity against anaerobes and 213 (83%) participants correctly answered that methicillin resistant *Staphylococcus aureus *(MRSA) is not susceptible to cephalosporins, the remaining participants (n = 41, 16%) incorrectly responded that it is susceptible to cefalotine, cefuroxime or ceftriaxone. The majority (n = 237, 93%) of participants agreed that amoxicillin is safe during the first three-month period of pregnancy whereas 17 (7%) incorrectly answered that ciprofloxacin or gentamicin are safe. A total of 180 (70%) participants correctly answered that ceftriaxone is the most effective drug crossing the blood-brain barrier where as 62 (24%) and 10 (4%) of participants incorrectly chose vancomycin and clindamycin above ceftriaxone. With regard to the estimate about local AMR rates, it was striking that only 51 (20%) of participants correctly estimated that > 50% of *K. pneumoniae *isolates are resistant to cephalosporins, whilst half, 129 (50%) answered that the resistance rate was 20%-50% and 47 (18%) answered 'don't know'. In response to the question about resistance rates of *P. aeruginosa *to ciprofloxacin, 82 (32%) of participants gave correct estimates (*i.e*. 20-50%), 118 (46%) answered that the rate was higher than 50% and 39 (15%) answered 'don't know'.

### Awareness about the current scope of AMR

**Figure 1:**
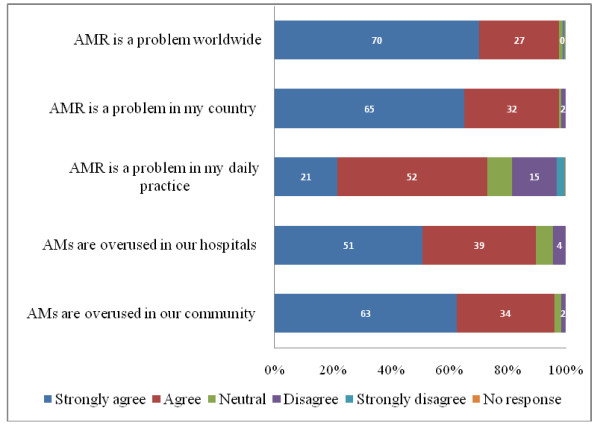
**Awareness of the scope of AMR among 256 participants (data in the graphs represent percentages)**. AM: Antimicrobial, AMR: Antimicrobial resistance

Almost all participants considered that AMR is a problem (98%). There were fewer residents than attending physicians who strongly agreed that AMR is a worldwide problem (58% versus 81%, p < 0.001). A similar scenario was observed in relation to the perception of AMR at the national level as 54% of residents strongly agreed that AMR was a problem compared to 78% of attending physicians (p < 0.001). However, whilst a mere 22% strongly agreed AMR is a problem in their own practice (Figure 1), Further, there was agreement upon the perception of overuse of AMs in both the Peruvian community and hospitals (96% and 90% combined "strongly agree" and "agree" answers for both settings respectively).

### Confidence and seeking of inputs

Nearly half (63/135, 47%) of residents revealed they were very confident about the optimal use of antimicrobials compared to 99/121 (82%) of attending physicians (p < 0.001). A total of 78 (31%) participants agreed that it is difficult for them to select the correct AM, this was recorded for 36% participants from the medical departments versus 20% from the surgical departments (p = 0.014). Moreover, it should be noted that almost a quarter of participants (n = 58, 23%), strongly agreed and agreed that prescribing AMs when they are not required does not cause any harm. With regard to seeking inputs, when participants were asked about the frequency of reviewing their decision to prescribe AMs with a senior colleague, 15% replied 'never' and 57% 'sometimes'; only 6% answered 'always'. More than half (74/135, 55%) of residents declared that they never or only sometimes reviewed their decision with a senior colleague compared to 89% (108/121) of attending physicians (p < 0.001). This was seen more frequently among participants from surgical departments compared to those from medical departments (80% versus 67%, p = 0.03). Among the 219 participants who declared to review their decision to prescribe AMs with a senior colleague at least sometimes, nearly three quarters (161, 74%) reported that senior colleagues sometimes recommended a different AM.

### Sources of information and continuing education about AMs

Overall, 88 (34%) participants declared that there had been no lectures about AM use as part of academic activities within their departments during the previous year, although there was a slight difference between the medical and surgical departments (29% versus 45%; p = 0.015). Likewise, 37% (95/256) of participants had not participated in a course on AM use during the previous year; the rate was 65% among residents versus 35% among attending physicians (p = 0.003). Regarding sources of information, two-thirds (173, 68%) of participants reported having readily available sources of information on AMs. The "Sanford Guide on Antimicrobial Therapy" was considered as a very useful source (n = 129, 50%), although preferentially among residents (n = 78, 58%) compared to attending physicians (n = 51, 42%, p = 0.013). Internet sources were considered as very useful or useful by nearly two-thirds (159, 62%) of participants. Thirty six (14%) participants did not consider the national guidelines useful and a quarter (65, 25%) noted that they were not familiar with these guidelines. Advice from colleagues of higher rank or same rank were considered useful or very useful in 98 (38%) and 71 (28%) of participants respectively.

### Factors influencing decisions around AM prescription

**Figure 2:**
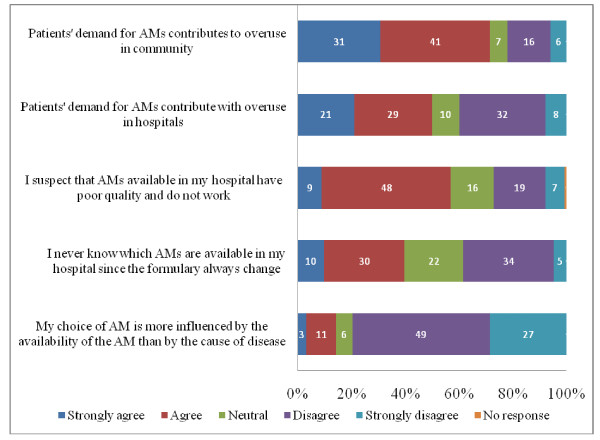
**Perception of factors influencing the decision on AM prescription**. Data represent percentages among 256 participants. AM: Antimicrobial

Nearly three quarters (183/256, 72%) of participants strongly agreed or agreed that patients' demand for AMs contributes to their overuse in the community, but only half (n = 128, 50%) did so for the hospital setting (Figure 2). Almost 40% (n = 102) of participants declared that they were unaware of the AMs available in their hospital because of continuously changing formulations. Surprisingly, more than half (146, 57%) agreed with the statement that the AMs available in their hospitals are of poor quality and are not effective.

### Acceptability and appropriateness of potential interventions

**Figure 3:**
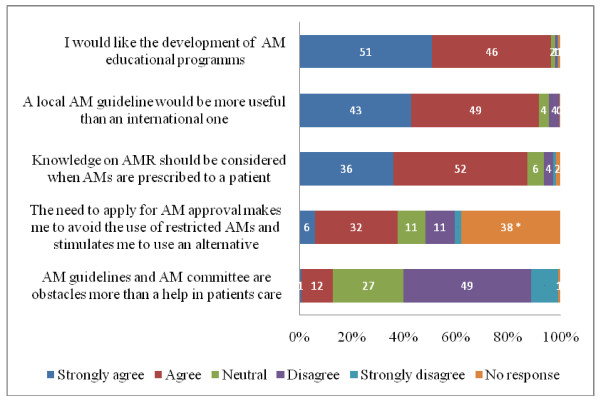
**Acceptability and appropriateness of potential interventions on AM prescribing as surveyed among 256 participants**. Data represent percentages. AM: antimicrobial, AMR: Antimicrobial resistance, *38% answered 'There is no a restrictive policy in my hospital'

The vast majority of participants strongly agreed and agreed with the development of AM prescribing educational programs (n = 247, 97%) and confirmed that a local AM guideline would be more useful than an international one (n = 235, 92%). Moreover, 224 (88%) participants strongly agreed and agreed that knowledge about local AMR rates should be considered when prescribing AMs. Ninety-six participants (38%) strongly agreed and agreed that the need to apply for approval to prescribe restricted AMs caused them to seek an alternative AM (Figure 3). More participants from Arzobispo Loayza hospital (88%) strongly disagreed or disagreed with the statement that AM guidelines and AM committees are an obstacle to patient care compared to participants from Cayetano Heredia hospital (45%) (p < 0.001).

## Discussion

The present study describes the results of a KAP-survey among 256 medical doctors (both residents in training and attending physicians) practicing in two large public teaching hospitals in the Lima area, Peru.

### Knowledge on AMs and AMR

Overall, the theoretical knowledge about AMs including indications, administration and side effects ranged from very good to excellent. Despite this apparently good score for these questions, it should be noted that a quarter of participants still considered that it was correct to use AMs for upper respiratory tract infections. This suggests that this issue should be targeted in future educational interventions. Furthermore, it is known that in practice AM use may not reflect these results, and this can be illustrated by a recent study: in a rural Peruvian village, 58% of children with acute upper respiratory symptoms or watery diarrhoea (for which AMs are not recommended) were given AMs when they went to see a doctor [12]. This contrasts with the survey's results, in which the majority of participants answered correctly that AMs are unnecessary for either of these conditions. Likewise, it should be noted that a quarter of participants agreed with the statement that unnecessary prescribing of AMs does not cause any harm. Although the participants' overall knowledge about AMs was appropriate, most of them incorrectly estimated the local resistance rates of two key-pathogens in the hospital setting, *K. pneumoniae *and *P. aeruginosa*. Similar findings have been described in other studies [7,8]: Pulcini *et al*. showed that only 16% of young doctors in a French hospital knew the actual proportion of community acquired-*Escherichia coli *resistant to fluoroquinolones. Local microbiology laboratories are encouraged to maintain a database about the levels of resistance of key pathogens and diffuse it to prescribers: when reinforced by the local antibiotic committee, information may orient prescribing doctors [13].

### Awareness about current scope of AMR

The awareness of AMR as a worldwide and national problem was very high among the participants. However, in contrast, AMR was much less recognised as a problem in participants' own practice. This trend has also been observed among physicians surveyed in the U.S. (Table 1) [9-11]. On the other hand, qualitative research among general practitioners in the U.S. showed that most of the physicians interviewed were aware that inappropriate use of AM in their own practice contributes to increasing AMR [14]. Interestingly, the majority of participants recognized excessive use of AMs as a factor contributing to AMR in the community, but only half did so for the hospital settings.

### Confidence and seeking of inputs

Compared to attending physicians, residents in training were less confident about AM prescribing. This correlates with the findings of Srinivasan *et al*.: in this study, senior residents were more confident about optimal use of antimicrobials compared with first year-residents [9]. Moreover, residents tended to seek advice from their senior colleagues when prescribing, irrespective of their specialization (department) or hospital affiliation, compared with attending physicians who have more years of clinical experience. However, more residents declared that they consulted internet-based sources rather than approaching senior colleagues for advice. It is surprising than more than 50% of residents declared that they did not consult senior colleagues considering that both institutions were teaching hospitals. Other sources of AM guidance are discussed below.

### Sources of information and continuing education about AMs

The present survey also revealed information about the sources of information for AM use. The popularity of the Sanford Guide illustrates the accessibility of pocket-based treatment guidelines. Internet sources were ranked as the second most useful source. In this scenario, distant learning technologies which have been used successfully in Peru for other disciplines [15,16] may have a place in promoting educational AM prescribing programs. The poor appreciation of and familiarity with the national guidelines among the participants is striking and contrasts with the seemingly large demand for local AM guidelines.

### Factors influencing decisions around AM prescription

Three quarters of participants identified patient demand for AMs as a key factor contributing to the overuse of AMs in the community, with half doing so for the hospital setting. Pressure from patients is indeed an important factor particularly in the middle- and low-income settings. A study among parents and paediatricians in Venezuela revealed that 87% of doctors felt pressured by parents into prescribing AMs; 48% of parents said that they had requested AMs and 33% revealed that they had obtained a prescription [17].

The high expectation about AM use from patients is very probably a consequence of their minimal understanding of AMR and AM side effects. Education of the general public through community-targeted media information is extremely important

More than half of participants agreed that AMs in their hospitals are of poor quality. Although we have not explored in detail the definition of "poor quality" according to the prescribers, there are several issues. Firstly, despite regional and national regulations for drug marketing, counterfeit (and probably substandard) drugs have been detected in Peru, but information was mainly distributed by the lay press and as such, it is difficult to estimate the magnitude of this problem. Secondly, in our experience, generic drugs are also frequently perceived to be less effective, an idea reinforced by recent studies from Colombia showing that generic vancomycin and oxacillin had a less therapeutic effect in animal subjects [18,19]. This is of concern, as a lack of confidence in generic and locally market drugs may similarly affect confidence in following standard treatment guidelines and in the implementation of essential drug lists and may deflect patients and prescribers towards the private sector. The Peruvian Ministry of Health should build confidence in the quality of locally available AMs by circulating adequate information about locally marketed AMs. In line with the need to diffuse data on AMR rates among key-pathogens, it is clear from the present results that the hospital pharmacy should diffuse timely and accessible information about the availability of AMs.

### Acceptability and appropriateness of potential interventions

Formal programs about AMR and AM prescribing were welcomed by the vast majority of participants suggesting a gap in knowledge about infectious diseases, microbiology and AM prescribing in university programs [20]. There was also strong agreement about the usefulness of local AM guidelines, although concerns about the acceptability of the local antibiotic committee and its steering measures should be addressed in the future.

One of the main limitations of KAP-surveys is the fact that participants may tend to give socially desirable answers rather than expressing their true opinions. The present setting of teaching hospital may contribute to this bias. In order to minimize this potential bias anonymous participation was ensured and the case-based questions about AM prescription (which might have been suggestive) were presented at the end of the survey. The fact that this survey was based on a survey conducted among U.S. physicians may be another limitation, but it was countered by the pre-release validation. In addition, the survey was extended to the local context by adding questions relevant to the Peruvian situation. Another issue was that physicians working in hospitals were also questioned about their knowledge and attitudes towards community infections. However, the majority of doctors in the two hospitals were practicing in both the hospital and the community setting. Further studies should be done to study the knowledge, attitudes and practice surrounding AM use among physicians from community centres. Finally, one may question whether the attitude of doctors in other parts of Peru to AMs is reflected by the results of this survey. As this study was conducted in two large, public, tertiary-level teaching institutions and involved a large number of prescribing doctors, we are confident that the results may be applied to other public general hospitals in Peru. However, the generalizability of the results to other health care settings remains to be demonstrated.

## Conclusion

The present KAP-survey has generated information about the prescribing attitudes and practices of medical doctors from public hospitals of a middle-income country. It identified topics to address in the containment of AMR, such as the dissemination of information about local AMR rates, the importance of renewing public confidence in the quality of locally available AMs, the revision and dissemination of local AM guidelines, addressing the general public and exploring the possibilities of internet-based trainings.

## Competing interests

The authors declare that they have no competing interests.

## Authors' contributions

CG designed the protocol and survey, obtained ethical approval, analysed the database and drafted the manuscript. LL, AJ and KG validated the questionnaire, collected data and completed the database. FS and EG assisted with preparation of research protocol and review of the manuscript. JJ collaborated with the preparation protocol and survey, analysis and redaction of the manuscript. All authors read and approved the final manuscript.

## Pre-publication history

The pre-publication history for this paper can be accessed here:

http://www.biomedcentral.com/1472-6904/11/18/prepub

## Supplementary Material

Additional file 1 **Knowledge, attitudes and practice survey about antimicrobial resistance and prescribing**. This is a 38-item questionnaire that evaluated the knowledge, attitudes and practice of antimicrobial use and antimicrobial resistance among physicians. Click here for file
