# Supplementary material for: Knowledge, attitudes and practice survey about antimicrobial resistance and prescribing among physicians in a hospital setting in Lima, Peru
Source: BMC Clin Pharmacol. 2011 Nov 15;11:18. doi: 10.1186/1472-6904-11-18 (PMC3231801; doi:10.1186/1472-6904-11-18)
Supplement: Additional file 1 — Knowledge, attitudes and practice survey about antimicrobial resistance and prescribing. This is a 38-item questionnaire that evaluated the knowledge, attitudes and practice of antimicrobial use and antimicrobial resistance among physicians. [file 1472-6904-11-18-S1.DOC]

**Knowledge, attitudes and practice survey about antimicrobial use and antimicrobial resistance**

**We want to thank you for receiving this survey. We hope this survey will help us to learn more and do a better job with antibiotic use in the hospital.**

**Please answer all questions as best you can and remember that all answers are anonymous.**

**First, we will start with general questions**

| **Nr** | **Question** | **Answer** |
| --- | --- | --- |
| 1. | After you left the medical school, how many years have you been working in a hospital? (if you have worked in two or more hospitals, please add the number of years in each hospital) |  1 year or less   2 years   3 years   4 years   5 years   6 years   7 years or more |
| 2 | Do you belong to which departments do? |  Medicine /Emergency   Surgery   Pediatrics  Obstetrics and Gynecology |
| 3. | Currently, what position do you have in the hospital? |  Resident   Attending physician |
| 4. | Do you think that knowledge of antibiotics will be important to you in your medical career (currently and in the future)? |  Yes   No |
| 5. | What do you think about antimicrobial resistance? |  It is a problem   It is not a problem   I do not know |
| 6. | How frequent do you prescribe antibiotics in the emergency room, outpatient clinic or in the wards? |  More than once a day   Once a day   3-5 times per week   1-2 times per week   Less than once a week |

**The following questions are about how you choose antibiotics**

| 7. | When you are in the emergency room, outpatient clinic or in the wards, how frequent do you review your decision to prescribe antibiotics with a senior colleague? |  Never   Sometimes   About half the time   Most of the time   Always |
| --- | --- | --- |
| 8. | If you ask to a senior colleague, how frequent he/she recommends you a different antibiotic? |  Never   Sometimes   About half the time   Most of the time   Always |
| 9. | How confident do you feel about the optimal use of antibiotics? |  Very confident   Somewhat confident   Somewhat unconfident   Unconfident |

**The following questions are about how you learn about antibiotics**

| 10. | During the last year, how many times have you received some teaching on antibiotics…? |  |
| --- | --- | --- |
|  | - As part of the academic activities of your department |  0   1-3 times   4-6 times   6-10 times   >10 times |
|  | - Participation of courses |  0   1-3 times   4-6 times   6-10 times   >10 times |
| 11. | Which of the following sources of information do you use as part of continuous medical education on antibiotics or when you have a specific question on antibiotics? Please check your rating in terms of its usefulness with 1= being very useful, 3 = useful and 5 = not at all. If you are not familiar with a source, please check that option |  |
|  | A) Information from senior colleagues | 1 2 3 4 5  Not familiar with |
|  | B) Information from colleagues of the same rank | 1 2 3 4 5   Not familiar with |
|  | C) Internet sources | 1 2 3 4 5   Not familiar with |
|  | D) Sanford Antimicrobial Guide | 1 2 3 4 5   Not familiar with |
|  | E) National guidelines for diagnosis and treatment in Peruvian hospitals | 1 2 3 4 5   Not familiar with |
|  | F) Others : please write it out  --------------------------------------- |  |
| 12. | Do you think you have enough sources of information about antibiotics when you need it? |  Yes   No, should be more sources of information (please specify which sources do you think would be useful): .................................. ...... |

**Tell us how strongly you agree or disagree with the following statements on antibiotics**

| 13. | Antibiotics are overused in my hospital and in other hospitals of my country |  Strongly agree   Agree   Neutral   Disagree   Strongly disagree |
| --- | --- | --- |
| 14. | Antibiotics are overused in the community in Peru |  Strongly agree   Agree   Neutral   Disagree   Strongly disagree |
| 15. | I believe it is difficult to select the correct antibiotic |  Strongly agree   Agree   Neutral   Disagree   Strongly disagree |
| 16. | Antimicrobial resistance is not a significant problem in my hospital |  Strongly agree   Agree   Neutral   Disagree   Strongly disagree |
| 17. | When I decide which antibiotic to use, my election is more affected by the expiration date/availability than the cause of the infection. |  Strongly agree   Agree   Neutral   Disagree   Strongly disagree |
| 18. | Patients’ demands for antibiotics contribute to overuse… |  |
|  | a- in inpatients |  Strongly agree   Agree   Neutral   Disagree   Strongly disagree |
|  | b- in outpatients |  Strongly agree   Agree   Neutral   Disagree   Strongly disagree |
| 19. | General knowledge on antimicrobial resistance should be considered when antibiotics are prescribed to an individual patient |  Strongly agree   Agree   Neutral   Disagree   Strongly disagree |
| 20. | The need to apply for antibiotic approval in my case made that I avoid the use of restricted antibiotics and try to find an alternative. If there is no a restrictive policy in your hospital, please check: No restrictions in my hospital. |  Strongly agree   Agree   Neutral   Disagree   Strongly disagree  No restrictions in my hospital |
| 21. | The development of a local guidelines would be more useful than the international ones |  Strongly agree   Agree   Neutral   Disagree   Strongly disagree |
| 22. | Antibiotic guidelines and antibiotic committee are an obstacle more than a help to clinical care |  Strongly agree   Agree   Neutral   Disagree   Strongly disagree |
| 23. | I would like the organization of educational programs on antibiotics |  Strongly agree   Agree   Neutral   Disagree   Strongly disagree |
| 24. | I never know which antibiotics are available in my hospital because the formulary always changes |  Strongly agree   Agree   Neutral   Disagree   Strongly disagree |
| 25. | I believe that prescribing antimicrobial does not cause damage when patients do not need them |  Strongly agree   Agree   Neutral   Disagree   Strongly disagree |
| 26. | I suspect that some antibiotics available in my hospital are of poor quality and for that reason do not work. |  Strongly agree   Agree   Neutral   Disagree   Strongly disagree |

**Tell us how strongly you agree or disagree with the following statements on antimicrobial resistance**

| 27. | Antimicrobial resistance is a problem worldwide |  Strongly agree   Agree   Neutral   Disagree   Strongly disagree |
| --- | --- | --- |
| 28. | Antimicrobial resistance is a problem in my country |  Strongly agree   Agree   Neutral   Disagree   Strongly disagree |
| 29. | Antimicrobial resistance is a problem in my daily practice |  Strongly agree   Agree   Neutral   Disagree   Strongly disagree |

**The following questions are related to the use of antibiotics and the rates of antimicrobial resistance**

| 30. | A 40 year-old woman went to the Emergency room complaining of 4 days of diarrhea (3 unformed stools per day). No history of fever. One month before she had a urinary tract infection and took ciprofloxacin. . Which antibiotic will you recommend? |  Ciprofloxacin   Trimethoprim-sulfamethoxazole   No need of antibiotic use. Oral rehydration. |
| --- | --- | --- |
| 31. | A 32 year-old male went to the clinic complaining of fever (39 ^o^C), nasal discharge and throat pain for 3 days. Which antibiotic will you recommend? |  Amoxicillin   Trimethoprim-sulfamethoxazole   Clarithromycin   No need of antibiotic use |
| 32. | During your stay in the ward, you have seen two patients with impaired kidney function. Patient A is a 68 year-old male with cellulitis in the lower limb. He received clindamycin. Patient B is a 64 year-old woman with diabetes who received empirically treatment for sepsis with ceftriaxone and gentamicin. In which case you will need to adjust the antibiotic dose?. |  patient A   patient B   patient A and  B   neither patient A nor patient B |
| 33. | Which one of the following antibiotics may be safe during pregnancy? |  Amoxicillin   Ciprofloxacin   Gentamicin |
| 34. | Which one of the following antibiotics has the best activity against anaerobes? |  Ciprofloxacin   Metronidazole   Trimethoprim-sulfamethoxazole |
| 35. | Methicillin resistant *- Staphylococcus aureus* is susceptible to: |  Cefalotin   Cefuroxime   Ceftriaxone   None of these antibiotics |
| 36. | Which one of the following antibiotic is more effective to cross the blood-brain barrier? |  Clindamycin   Ceftriaxone   Vancomicin |

| 37. | The rate of resistance of *Klebsiella pneumoniae* to cephalosporins in your hospital is …. |  >50%   20%-50%   20%   I do not know |
| --- | --- | --- |
| 38. | The rate of resistance of *Pseudomonas aeruginosa* to ciprofloxacin in your hospital is …. |  >50%   20%-50%   20%   I do not know |

**Thank you very much for filling this survey**
